# The impact of culture perception on kinship disconnection of Chinese youth: examining the chain mediating effect of kinship support, kinship burnout, and social media interaction

**DOI:** 10.3389/fpsyg.2023.1226742

**Published:** 2023-11-30

**Authors:** Ruixia Han

**Affiliations:** ^1^School of Media and Communication, Shanghai Jiao Tong University, Shanghai, China; ^2^Institute of Cultural Innovation and Youth Development, Shanghai Jiao Tong University, Shanghai, China

**Keywords:** breaking off kinship, culture perception, kinship support, kinship burnout, kin interaction size, kin group activity

## Abstract

Kinship connections are an essential foundation of social relationships in China, yet in recent years there has been an increasing tendency toward kinship disconnection (Duanqin) among Chinese youth. In this study, based on online survey data of 555 Chinese individuals aged 18–35 years under a comprehensive explanatory framework of culturalism, functionalism, and behaviorism, regression and mediation analyses found that (1) cultural perception based on family-state values is the dominant factor influencing people’s tendency to disconnect. (2) Kinship support decreases people’s propensity to break off relatives, while kinship burnout increases people’s propensity to cut off relatives. Both act as functionalist factors in the chain that mediates the effect of cultural perception on the propensity to break kinship, and kinship burnout obscures the effect of kinship support and becomes a differentiating variable. (3) Both social media kin interaction size and kin group activity significantly reduce people’s propensity to disconnect. However, group activities are more significant in cultural perception’s mediating effect on disconnection propensity. It indicates that truly “active” social media connections are more important. The study attempts to propose a framework of “cultural perception + functional satisfaction and burnout + social media” to interpret Chinese youth kinship communication activity. On a practical level, more support for young people in social media interactions could slow or even reverse the trend of disconnection.

## Introduction

In recent years, more young people in China are becoming estranged from their families. A study of young people showed that 77.22% of 18–25 year olds only “occasionally” or “never” contact their relatives, and 71.11% of 26–30 years olds (Hu and Han, 2022). This phenomenon has also received media attention. For example, after the 2023 Chinese New Year, major Chinese newspapers such as Southern Weekend and The Beijing News, in addition to online media source, The Paper (Pengpai), and social media outlets Douban, Bilibili, Tiktok or Kuaishou, all published articles and reports covering the topic of Chinese youth becoming estranged from their family (Liu, 2023; Yg, 2023). One video article on Bilibili, “The Wave of Kinship Disconnection Is Sweeping China’s Younger Generation,” reached nearly 1.06 million views (Hardcore, 2023). Many phenomena show “kinship disconnection” (Duanqin) forming among Chinese youth. “Kinship disconnection” refers to the phenomena of when young people, who are tired of kinship ties, adopt an attitude of rejecting or refusing to partake in interpersonal communication with their family, or even directly “cutting off ties” with their family members (Hu and Han, 2022). There are three stages which comprise the phenomena of Kinship disconnection: alienation, dilution, and distancing (Hu and Han, 2022). Previous literature has sought to explain the growing rates of young individuals’ estrangement with their families by investigating the impact of urbanization upon young Chinese individuals (Yan, 2021; Hu and Han, 2022). However, the trend of urbanization has been in effect since 1992 in China (Gao et al., 2015), and the recent rise in kinship disconnection has taken place within the last few years.

An examination of the phenomenon of kinship breaking need to review and analyze Chinese family traditions and organizational structures. In fact, family relations have always been an important foundation for the functioning of Chinese society (Giskin and Walsh, 2001; Chu, 2010). “Family” is also considered by many Western scholars to be an important lens for understanding Chinese culture (Ebrey, 1991). As early as 1948, Chinese sociologist Fei Xiaotong proposed the theory of “differential modes of association”(Chaxugeju), which refers to the construction of Chinese social relations as a ripple of individual-centered, parents, siblings, relatives and geo-relative relations expanding outward (Fei et al., 1992). This is closely aligned with traditional Chinese agrarian culture. By the 1980s in China, this support of kinship ties of family group played an important role in the transformation of modern enterprises in South China, and it even became support for the modernity of the late 20th century (Weidenbaum, 1996; Redding, 2000). It means the family is an important resource for the social life of individuals, both in terms of the traditional agrarian way of life and in terms of the modern post-factory mode of organizing social life (Yan, 2021). Therefore, it is not difficult to understand why some Chinese scholars still consider “home” as the main cultural and organizational resource for seeking social integration and functioning (Xiao, 2017).

For the past few years, Chinese youth really seem to be tired of “family” and have a tendency to “cut off relatives,” especially during the Chinese New Year, a traditional Chinese family reunion ritual. As China’s socio-economic transition continues, the Chinese New Year has taken on a new economic and ritual significance (Xiao et al., 2017). The lack of return home or the active avoidance and denial of contact with relatives during the Chinese New Year is a further indication of the seriousness of this problem. Different scholars have given different explanations, for example, Xiang (2021) thinks modern families cannot be too aggressive and affect personal development, which actually involves the impact of family and personal boundaries also known as family negativity. Hu and Han (2022) believes that social comparison and pressure brought by extended family connection as well as changes in social structure are the reasons for the formation of the trend of family disconnection, and Sun (2023) summarizes that there are three major perspectives to interpret this phenomenon: moral and cultural perspective, class mobility perspective and social relationship perspective. That is, people focus on the structural level to find the causes, but what causes or perspectives can lead to a more comprehensive analysis from the individual level of analysis?

This paper intends to analyze kinship disconnection by Chinese youth in three theoretical approaches: culturalism, functionalism, and behaviorism. Culturalism focuses on the role of culture in people’s daily practices and lifestyles from the path of British cultural studies, which emphasizes the influence of soft factors such as meaning and values more than structuralism factors (Dirlik, 1987). In our study, the main focus is on whether family values specific to China influence people’s choice of kinship or kin-breaking behavior (Xu and Xia, 2014). Functionalism is mainly from the perspective of structural functionalism since Parsons, that is, certain cultural settings and relational structures exist because they fulfill certain functions (Swenson, 2004). For the phenomenon of kinship disconnection among Chinese youth, is it because kinship connections not only do not give enough support but also bring burnout that eventually contribute to this “rational” choice of theirs? For the behaviorism perspective, which focuses on the objective process of behavior (Muchon de Melo and de Rose, 2013), with the internetization of social behavior, we are concerned whether the scale of kinship connections and the status of interactions in social media can predict their “disconnection” behavior. These three theoretical perspectives basically encompass the possible factors from “agency” to “structure” and inspire us to propose a combination of variables to explain the phenomenon. From a comparative perspective, unlike Xiang, Hu, and Sun’s explanations above, this theoretical framework is more suitable for explaining kinship choices made from the standpoint of young actors in contemporary social contexts. Corresponding to the existing studies, our research questions thus focus on whether the tendency of Chinese young people to disconnect is influenced by the cultural conception of family-state views that may be closely related to the issue, and whether family support or burnout of the individual affects the tendency to disconnect. Finally, from the perspective of behavioral prediction, are the scale and frequency of kin interactions on social media a valid perspective to observe the tendency of young people to disconnect? What are the patterns of relationships between functional, behavioral, and cultural variables? By answering the above questions, we hope to have a comprehensive explanation of the current phenomenon of kin disconnection among Chinese young people and to dialog with the existing research explanations.

## Literature review and research hypothesis

### Cultural perception: the possible influence of family-state view on Chinese youth’s tendency to break off kinship

Cultural value plays a critical role in people’s relationships with kin (Hsu, 2017). Diverse cultural forms are closely related to the value of kinship, and according to Schneider, “on a purely cultural level, there is no such thing as kinship” (Sahlins, 2011). Within this pattern of relationships, different cultures and countries have developed different family sizes and kinship relationships (Schwartz, 2006). For China, the distinction between “ethical-based structure” and “individual-based structure” in Liang Shuming’s analysis (1949) of the cultural differences between East and West has been an essential concept in the exploration of the cultural characteristics of the Chinese family (Weiming and Xiaoyu, 2009). In a modern sense, different cultural types affect family size, how family members are connected, and the raising of offspring. For example, Popenoe analyzed the changing structure and patterns of the Western family (Popenoe, 2020), while Selin analyses different approaches to parenting in non-Western cultures (Selin, 2013), Ponczek et al. directly analyses the causal effect of family size on child quality in a developing country (Ponczek and Souza, 2012). Regarding externalities, family structures may influence economic growth and development patterns (Greif, 2006). Not only do different cultures correspond symbiotically to different family structures and values, but in turn different types of family structures also influence the logic of intergenerational parenting and the economic performance of the societies in which they operate.

“The differential mode of association” is a fundamental concept for understanding interpersonal relationships in Chinese society. This concept, proposed by Fei Xiaotong in 1948, points out that the social relations of Chinese people based on the vernacular foundation of agricultural society are characterized by a gradual outward extension from parents, relatives, and kin (Barbalet, 2021). Although it is said to be village-centric, the “relationship” (Guanxi) concept subsequently developed by Bian Yanjie and others on top of this has become a key to understanding modern Chinese society (Bian, 2018). Furthermore, changing cultural attitudes also influence changes in family structure and size (Coombs and Sun, 1978; Popenoe, 2020). This is especially true when the composition of family size affects the distribution of resources within the family (Blake, 2022). However, the sequential unity of the family state in Eastern cultures, especially Confucianism, and its dominant role in the functioning of modern social organization encourage young people to maintain ties with their original kin and reduce disconnection or tendency to disconnect, so we hypothesize that.

*Hypothesis 1*: cultural perception of the “family state” is negatively associated with young people’s tendency to disconnect.

### Functionalism perspective: the role of kinship support and kinship burnout in the development of Chinese youth’s tendency to break off kinship

In fact, with the transformation of Chinese society and its development on modernization, scholars have increasingly found that the simple cultural concept of “the differential mode of association” is no longer sufficient to explain the interpersonal networks of the Chinese public after the 1990s, which led Li Peiliang to propose the concept of “instrumentalist differential order pattern” (Peiliang, 1993) which points out that the differential pattern based on blood is insufficient to explain the relationship connection of people in modern society based on work units and organizations, and that the social organization model close to the Western “individual” connection will replace the original “ethics” based kinship connection model. Wellman (2002) demonstrates that in the functioning of modern urban-centered societies, non-territorial and loosely based connections supported by specialization characterize the relational connections of urban populations. So what kind of people will be central in forming new relational networks? Granovetter’s research on social relations provides extensive support towards answering this question (Granovetter, 1973). Granovetter’s findings suggest that those who are able to provide relatively strong social capital support will become significant figures in an individual’s social network, while those who cannot will be on the periphery. Overlaying Chinese sociocultural attributes, the researchers Bian and Ang found that episodic “ties” based on kinship and geography remain variables that support people in important life events such as when searching for employment (Bian and Ang, 1997). Granovetter’s and Bian’s studies point to the emotional and material support that traditional kinship ties can provide in terms of relationships, networks, and information support, that is, a gradual convergence with the concept of social capital. Prandini’s research points out that the family is an important source and building block of social capital (Prandini, 2014). We hypothesize that a young person may be less inclined to disconnect if he or she receives more material, emotional, informational and network support from kinship.

*Hypothesis 2*: Kinship support is negatively associated with young people’s tendency to disconnect.

However, it is important to acknowledge that while families can support individuals, they can also be a hindrance or even be harmful, sometimes in the form of material, emotional, and psychological burdens. Ennis and Bunting’s research has found that family burdens can have an impact on people’s quality of life, often in the form of mental health responses (Ennis and Bunting, 2013), while significant stigmatizing events within the family can have an even more devastating impact on a person’s mental health (Lefley, 1989). Some different approaches and classifications have been proposed for the measurement of burden. A distinction between objective and subjective burdens prevails (Schene, 1990), which has evolved to be described directly by the term “perceived family burden”. In perceived family burden, “use abusive language,” “blame others,” “hardly talk,” and “act suspicious” were all measured (Levene et al., 1996), meaning that any speech or behavior from within the family can be a source of burden. When this family damage spreads to the kin level, it may significantly impact the individual and even produce family burnout (Ensle, 2005). In the case of Chinese families, extended families or familial families based on “kinship networks” can extend the range of sources of victimization. Especially after the modern transition, when young people generally develop an individualistic self-concept, people are likely to become more intolerant of various types of “harm” from different relational distances and to resort to kinship disconnection. We therefore hypothesize that.

*Hypothesis 3*: kinship burnout is positively associated young people’s tendency to disconnection.

### Behaviorism perspective: the effect of kin network interaction in social media on Chinese youth’s tendency to break off kinship

Many studies have argued that the increased geographical distance of young people who have left home for school or work after modern society’s transformation will dilute people’s kinship connections. In reality, however, the widespread use of social media today makes it easier for people to connect daily. At the same time, even people in close proximity may interact online, i.e., people’s daily interactions and communication behaviors are generally being mediated by the Internet (Yus, 2011). In this context, we can look for more objective indicators when considering the impact of online kinship interactions on the tendency to disconnection. As early as 1976, Mayhew and Levinger analyzed the effects of the size and density of population interactions on patterns of urban population association (Mayhew and Levinger, 1976). In contemporary analyses of people’s social networks, interaction size and frequency have been variables considered, and they affect the structure of individual social networks (Hill and Dunbar, 2003). Many studies on online interactions have also shown that interaction size and frequency do have an impact on family relationships (Subrahmanyam and Greenfield, 2008). It has also been found in everyday life that people who usually have larger and more frequent contact with family members and relatives are also less likely to exhibit sudden disconnection behaviors. We therefore hypothesized:

*Hypothesis 4*: the scale of kinship interaction is positively associated with young people’s tendency to disconnect.

The emergence of social media provides a new platform for people to connect one-to-one daily. It also allows group connections to be reproduced or developed in the form of various “groups,” people can reconstruct groups in their daily lives on social media. For young Chinese people, various groupings are essential to their social media platforms. Traditional family groups based on blood and marriage also appear in different forms in their social media contact lists. The “differential pattern” can be realized in the group matrix of social media platforms and various combinations oriented by relational closeness and relational needs. Just as the scale and frequency of interactions with relatives in daily life can predict whether young people will engage in disconnection behaviors, the daily activities in various family groups should, to some extent, explain or predict the tendency of young people to connect with relatives in terms of relational closeness, so we hypothesize that:

*Hypothesis 5*: the activity of family group interactions is positively associated young people’s tendency to disconnection.

### The interaction of three perspectives: kin support and burnout, and the role of social network interaction in the influence of cultural perception on young people’s tendency to break off kinship

The above literature demonstrates that young people with stronger cultural perceptions of a family may be less likely to engage in family disconnection behaviors, while kinship support and burnout negatively and positively affect young people’s tendency to disconnect, so what kind of relationship exists between the three? In fact, for traditional Chinese family relationships, cultural perception has always been the dominant influence, and Fei Xiaotong, in his “From the Soil: The Foundations of Chinese Society,” treats the benefits of this model of family union as a set of “chopsticks” that can play more than a chopstick’s role (Gu et al., 2017). In such cases, it sometimes even happens that family needs are met at the expense of personal interests. This is also considered proof that traditional Chinese society is based on “collectivism” rather than “individualism,” and since the 1970s, Granovetter’s research on strong and weak relationships has entered the field of Chinese social phenomena, combining with the original research concepts to form a unique field of “relationship” research. The field of “relationship”(*Guanxi*) research is unique. This field began to focus on introducing some of the measurement dimensions of strong and weak ties research, namely the variables related to social capital: material, emotional, information. Li Peiliang then developed the concept of “instrumental” differential patterns. Since there is support, there must be damage, and the damage will be around “emotion,” “material,” “relationship,” etc. Thus, it is reasonable to infer that although cultural perception may still dominate people’s propensity for kinship connections, kinship support positively strengthens such relationships, and kinship burnout negatively reduces them, so we hypothesize that:

*Hypothesis 6*: kinship support will strengthen the negative effect of cultural perception on young people’s tendency to disconnect.

*Hypothesis 7*: kinship burnout will weaken the negative effect of cultural perception on young people’s tendency to disconnect.

Indeed, any relationship’s maintenance relies on maintaining people’s dynamics, and ongoing interactions reinforce the recognition and maintenance of shared values (Bayer et al., 2016), and poor interactions can be disruptive (Aldridge, 1984). In general, the scale of daily interactions reduces the likelihood that people will suddenly break these ties in the first place because it means that individuals have been cultivating resources in this social network. A sudden break may result in the loss of previous social capital accumulation (Ellison et al., 2014). Therefore, it is reasonable to hypothesize that populations with a large scale of daily kin interactions enhance the influence of cultural perceptions on the tendency to maintain kin-connected relationships and reduce the propensity or likelihood of people disconnecting. It should be noted that in today’s world, where social media fully mediates people’s interpersonal relationships in daily interactions, this interpersonal scale is more appropriately measured by the social media interaction kinship scale. At the same time, the activity status of kinship groups based on different affinities in social media is a better predictor of individuals’ tendency to break kinship. Intra-group activities are essential for members to maintain relationships (Bryant and Marmo, 2009). Tong and Walther also showed that many family groups enhance connections precisely through group activities (Tong and Walther, 2011). Moreover, frequent in-group activities may further enhance the influence of cultural perception on people’s kinship maintenance, acting as a positive moderator or mediator. We, therefore, hypothesized that:

*Hypothesis 8*: the size of kinship interactions will strengthen the negative effect of cultural perception on young people’s tendency to disconnect.

*Hypothesis 9*: the activity of family group interactions will strengthen the negative effect of cultural perception on young people’s tendency to disconnect.

Based on the above research hypotheses, we further consider how functionalism variables such as family support and burnout connectively contribute to young people’s propensity to break off kinship. Similarly how do behaviorism variables such as interaction size and interaction frequency connectively contribute to young people’s propensity to break off kinship? We therefore continue to formulate the following hypothesis:

*Hypothesis 10*: Kinship support and burnout have chain mediating effect of cultural perception on young people’s tendency to disconnect.

*Hypothesis 11*: The scale of kinship interaction and group activity have chain mediating effect of cultural perception on young people’s tendency to disconnect.

## Method

### Sample and data

This study used an online survey to collect data related to kinship contact status of 555 young people aged 18–35 years from 30 provinces and cities in mainland China (data were not collected from Qinghai and Xinjiang) from February 6 to February 14, 2023. We used the sample service of Questionnaire Star,^1^ and at the same time, we made some controls. For example, access paths, number of answers, and logical questions are set so that a phone number can only be registered once and cannot be answered repeatedly, as well as limiting the number of times a user can break off and resume answering. The final demographic data we recovered for the sample is as follows: 87.7% had a bachelor’s degree, and 68.4% received 5,000–14,999 CNY per month. Married people accounted for 62.5%, and the average age was 28.6. In conjunction with the study topic, the place of birth and the relationship with the current place of work of the survey respondents were also examined, as detailed in Table 1.

**Table 1:** Distribution of the sample’s socio-demographic information (*N* = 555).

	Categories	Frequency	Percentage (%)
Gender	Male	258	46.5
	Female	297	53.5
Education	High school and below	11	2.0
	College/University	487	87.7
	Master	52	9.4
	Doctor and above	5	0.9
Income (CNY/Month)	0	29	5.2
	<4,999	70	12.6
	5,000–9,999	230	41.4
	10,000–14,999	150	27.0
	15,000–19,999	47	8.5
	20,000–24,999	18	3.2
	=>25,000	11	2.0
Married	Single	205	36.9
	Married	347	62.5
	Divorce	3	0.5
Birthplace	City	329	59.28
	Town	53	9.55
	Rural	173	37.17
Work/study place same with birthplace	Yes	292	52.61
	No, different cities or counties in the same province	177	31.89
	No, other provinces	86	15.5
Age	Mean	28.6	

### Measures

#### Tendency to break off kinship

For kinship relationships, festivals are the most testing time for people’s connection density (Humphrey, 1979). Furthermore, for Chinese people, Spring Festival is the biggest festival where people talk about “reunion” and even relatives who are not usually in contact with each other usually have a chance to meet (Xiao et al., 2017). Therefore, we used the question of who we spent the Spring Festival with to measure our tendency to break off kinship. The specific question is: How did you spend this Spring Festival? The multiple-choice items are 1. Attended multiple gatherings or visited multiple relatives; 2. Only visited or participated in gatherings with 1–2 relatives; 3. Only visited or spent time with parents (or spouse’s parents); 4. Spent time by myself or small family and did not visit or participate in gatherings with other relatives; 5. Spent time with friends and did not visit or participate in gatherings with other relatives or others. The tendency to break off the kinship variable has a maximum value of 5 and a minimum value of 1 (M = 1.77, SD = 1.004). It is important to note that for tendency to break off kinship perhaps a more multidimensional measure would be more meaningful, as some researchers have done for similar cultural concepts (Shi and Wang, 2019), and we will follow up by deepening our measurement of this concept.

### Cultural perception (view of family-state)

As Weiming and Xiaoyu (2009) believed 100 years ago, Chinese people have an “ethical-based structure” of cultural values instead of Westerners’ “individual-based structure.” (Weiming and Xiaoyu, 2009). The emphasis on “state” and “family” has become an essential indicator of the extent of traditional Chinese values. Combining the sections on “state” and “family” in Kim et al.’s measure of Asian cultural values (Kim et al., 1999) and the question items in Shi (1999) measure of Chinese cultural values, we ended up with the following measurement questions: Please give your opinion on the following views: 1. children should be filial to their parents; 2. kinship in the extended family is the basis of one’s social relationship; 3. there is a family before there is a country, and a well maintained extended family is the guarantee of national stability. The options are 1. strongly agree; 2. relatively agree; 3. generally agree; 4. relatively disagree; 5. strongly disagree. The answers are assigned in reverse order, added together, and divided by 3 to obtain the indicator “View of family-state” (Cronbach’s *α* = 0.721, M = 4.06, SD = 0.76).

### Kinship support

We draw primarily on Granovetter’s discussion of social capital and social support to measure kinship support (Fulkerson and Thompson, 2008). With the characteristics of people’s interpersonal connections in the Internet era (Carr et al., 2016), the support that young people can access in their family networks was classified into four categories: material, emotional, informational, and relational. The measurement question was as follows: please rate your overall interaction with the following categories of people. The vertical categories include parents, immediate siblings and grandparents, and relatives. The horizontal scale includes 1. receiving material or monetary help; 2. receiving advice, opinions, and emotional support; 3. receiving information or network support that is useful for your study or work. The matrix options are always, often, occasionally, and none, and after assigning 4, 3, 2, and 1, respectively, they are summed and divided by 9 to obtain the kinship support indicator (Cronbach’s *α* = 0.768, M = 2.69, SD = 0.51; Min = 1.22, Max = 4).

### Kinship burnout

Regarding the measurement of kinship burnout, we mainly referred to the measurement of burnout and family burden (Levene et al., 1996), combined with the problems that often occur in Chinese people’s life, such as the sense of boundary, and finally, locked in the following four items: 1. often compare you with others; 2. often ask questions that you do not like to answer; 3. have done something terrible to you; 4. bring trouble to you and add affairs. The vertical categories still include parents, immediate siblings, grandparents, and relatives. The matrix options include always, often, occasionally, and not. After assigning the values 4, 3, 2, and 1, the summation is divided by 12 to obtain the family burnout indicator (Cronbach’s *α* = 0.859, M = 3.17, SD = 0.53; Min = 1.08, Max = 4).

### Scale of closely interacted relatives

For the measurement of the size of closely interacted relatives, we used the following question: the number of people you interact with most closely in your WeChat list (including private chat, group chat, likes, comments, and retweets) is three types of people in the vertical matrix: parents, immediate siblings and grandparents, and relatives. The horizontal selection matrix options include 1.0 people; 2.1–2 people; 3.3–7 people; 4.8–15 people; 5.16 and above. The size of the closely interacted relatives indicator was obtained by adding the scores of each option and dividing by 3 (M = 2.51, SD = 0.65).

### Activity in family groups

Our measurement question for activity in family groups was: What is your membership in various family groups? The vertical column includes: 1. groups with parents; 2. groups where the primary members are parents, immediate siblings, and grandparents; 3. groups where the main members are (cousins, cousins) siblings; 4. extended family groups of relatives. The horizontal coordinates include: 1. have and often speak and interact; 2. have but rarely speak; 3. have but hardly speak; 4. have not; 5. once had and have disbanded or withdrawn from the group. The options were assigned 0–4 in reverse order, and the results were summed and divided by 4 to obtain the family group activity index (Cronbach’s *α* = 0.768, M = 2.68, SD = 0.77; Min = 0.22, Max = 4).

### Statistical analysis

**Figure 1:**
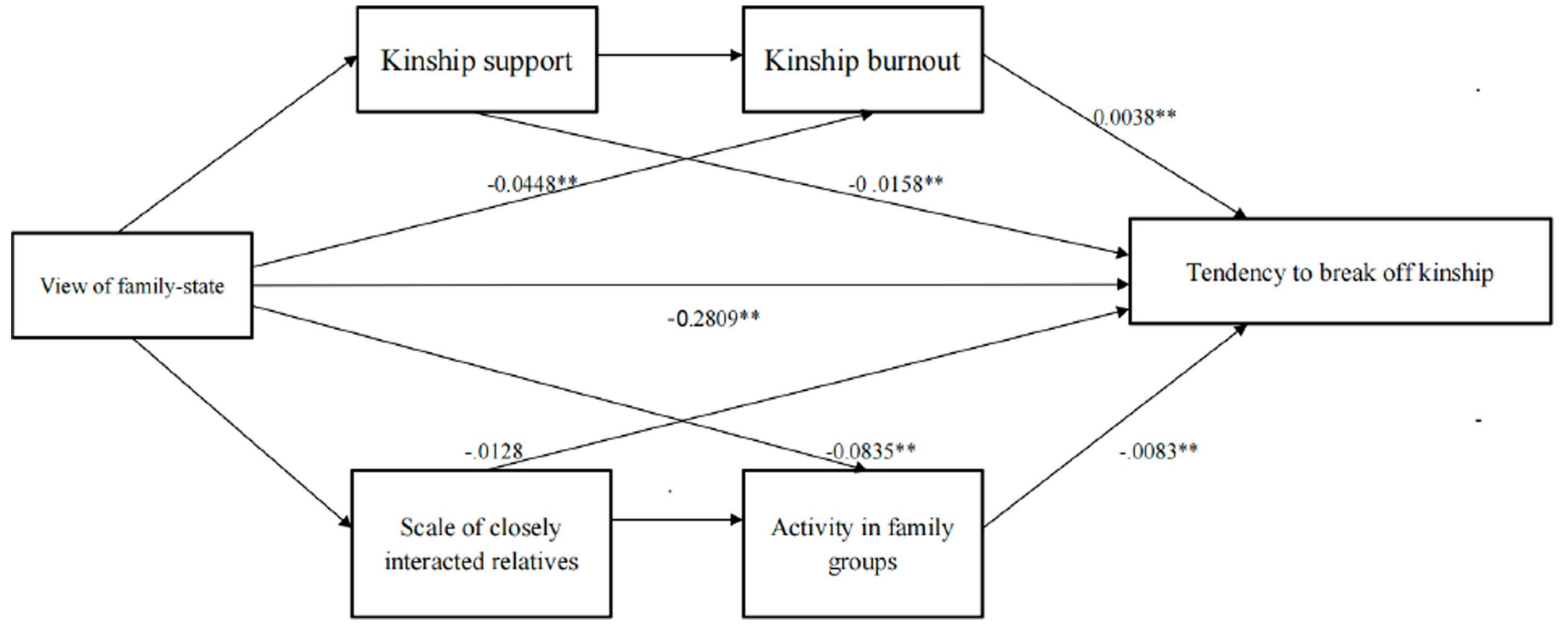
“Culture perception + ‘support-burnout’ + interaction” effect relationship on tendency to break off kinship. **p<0.01.

We examine the mediated effects of different combinations of variables by using model 82 in the PROCESS macro (Hayes, 2018) to answer h1–h5. The regression results are summarized in Table 2. Further, the chain-mediated results are summarized in Table 3 and Figure 1 to answer h6–h9 and RQ1 and RQ2.

**Table 2:** Results of regression analysis of tendency to break off family (*N* = 512).

	Kinship support	Kinship burnout	Size of closely interacted relatives	Activity in family groups	Tendency to break off kinship	Tendency to break off kinship
Constant	1.835** (8.394)	2.271** (10.028)	1.074** (3.622)	1.482** (4.505)	3.873** (9.973)	4.404** (9.891)
Male	0.109* (2.348)	−0.041 (−0.906)	0.053 (0.970)	−0.062 (−1.034)	−0.031 (−0.378)	0.013 (0.163)
Age	−0.010 (−1.326)	0.025** (3.529)	0.005 (0.623)	0.025** (2.645)	0.009 (0.714)	0.006 (0.455)
Education	0.025 (0.402)	−0.137* (−2.310)	0.006 (0.087)	−0.111 (−1.424)	−0.089 (−0.815)	−0.066 (−0.639)
Income	0.041 (1.828)	0.003 (0.137)	0.009 (0.348)	0.037 (1.265)	−0.055 (−1.364)	−0.031 (−0.808)
Married	0.126* (2.154)	−0.135* (−2.363)	0.167* (2.435)	0.049 (0.651)	−0.130 (−1.252)	−0.005 (−0.052)
Value of family-state	0.056 (1.822)	−0.198** (−6.675)	0.039 (1.043)	0.256** (6.298)	−0.425** (−7.842)	−0.280** (−4.996)
Kinship support		0.256** (5.918)	0.463** (8.665)	0.459** (7.326)		−0.219* (−2.504)
Kinship burnout			−0.014 (−0.266)	−0.270** (−4.635)		0.221** (2.791)
Scale of closely interacted relatives				0.214** (4.383)		−0.134* (−2.030)
Activity in family groups						−0.242** (−4.084)
*R* ^2^	0.045	0.150	0.185	0.332	0.129	0.222
Adjusted *R*^2^	0.034	0.138	0.172	0.320	0.119	0.206
*F*	*F* (6,505) = 3.962, *p* = 0.001	*F* (7,504) = 12.692, *p* = 0.000	*F* (8,503) = 14.277, *p* = 0.000	*F* (9,502) = 27.767, *p* = 0.000	*F* (6,505) = 12.480, *p* = 0.000	*F* (10,501) = 14.277, *p* = 0.000

*p < 0.05 and **p < 0.01, *T*-value in parentheses.

**Table 3:** Summary of process model 82 mediation test results.

Items	Indirect effect	BootSE	BootLLCI	BootULCI	Direct effect	Test results
Total indirect effect	−0.1615**	0.0358	−0.2360	−0.0986		
VFS = >kinship support = >TBOF	−0.0158**	0.0110	−0.0420	−0.0001	−0.2809**	Partial mediation effect
VFS = >kinship burnout = >TBOF	−0.0448**	0.0171	−0.0807	−0.0144	−0.2809**	Partial mediation effect
VFS = >SCIR = >TBOF	−0.0128	0.0085	−0.0327	0.0007	−0.2809**	No significant mediation effect
VFS = >AFG = >TBOF	−0.0835**	0.0246	−0.1350	−0.0398	−0.2809**	Partial mediation effect
VFS = >kinship support = >kinship burnout = >TBOF	0.0038**	0.0026	0.0001	0.0102	−0.2809**	Masking effect
VFS = >SCIR = >AFG = >TBOF	−0.0083**	0.0051	−0.0198	−0.0002	−0.2809**	Partial mediation effect

**p < 0.01. TBOF, tendency to break off kinship; VFS, view of family-state; SCIR, scale of closely interacted relatives; AFG, activity in family groups.

## Results

### Significant variables influencing young people’s tendency to break off kinship

The final model in Table 2 shows that demographic variables such as gender, age, education, income, and marital status do not significantly affect young people’s propensity to disconnect compared to the core variables we focus on. In contrast, cultural perception, kinship support, burnout, social media kin interaction size, and kinship group activity all significantly influence the formation of young people’s propensity to disconnection. For example, in the overall regression model, the cultural perception had the highest influence coefficient of *β* = −0.280 (*p* < 0.01), which means that the stronger the family-state values are, the less likely young people are to develop the tendency of family disconnection, supporting H1; kinship support acquisition showed a negative correlation with young people’s tendency of kinship disconnection, with an influence coefficient of *β* = −0.219 (*p* < 0.01), which means that the more kinship support they receive, the less likely they are to develop the tendency of kinship disconnection, supporting H2; while conversely young people with greater kinship burnout are more likely to break off kinship (*β* = 0.221, *p* < 0.01), supporting H3. Regarding social media kinship connections, the size of closely interacted relatives showed a significant negative correlation with a tendency to break off kinship (*β* = −0.134, *p* < 0.05). Youth family group activity also showed a significant negative correlation with a tendency to break off kinship (*β* = −0.242, *p* < 0.01), demonstrating that youth with large size of daily social media kin connections are less likely to tend to break off kinship. Youth with high activity in social media kin groups are also less likely to have less tendency to break off kinship tendencies, demonstrating that H4 and H5.

### The mediating role of kinship support and burnout, social media interactions in the relationship between cultural perception and tendency to breaking off kinship

The results of the chain mediation effect test using the process model 82 showed that *R* = 0.4686, *R*-sq = −0.2196, *F* = 28.4763, df1 = 5.00, df2 = 506 0.00, MSE = 0.7441, and *p* = 0.0000, indicating that the basic model fits well and that the model and the results can be adopted. Further interpretation of the results shows that the direct effect of a view of family-state on a tendency to break off kinship is −0.2809 (se = 0.550295, *t* = −5.0573, *p* = 0.0000), and the effect result does not contain 0 (LLCI = −0.3900, ULCI = −0.1718). Meanwhile, the overall indirect effect was also significant, with an effect size of −0.1615. Analysis of the chains of influence revealed that: (1) Kinship support strengthened the negative effect of cultural values on young people’s tendency to break off family, with a partially mediated effect occurring, with an effect size of −0.0158 (LLCI = −0.0420, ULCI = −0.0001), as evidenced by H6. (2) Kinship burnout partially mediated the negative effect of cultural perception on the tendency to disconnection, with an effect value of −0.0448 (LLCI = −0.0807, ULCI = −0.0144). H7 was demonstrated (3) Social media kinship interaction size did not significantly mediate cultural perception’s effect on the tendency to disconnection (LLCI = −0.0327, ULCI = 0.007). H8 was not demonstrated. (4) Kin group activation played a significant mediating effect in the effect of cultural perception on the tendency to break kin (LLCI = −0.1350, ULCI = −0.0398) with an effect value of −0.0385, as evidenced by H9. Further examination of the chain mediating effect of kinship support and burnout reveals that the positive effect of kinship burnout on the tendency to break off kinship obscures the negative effect of kinship support, with the chain ultimately producing a positive effect value of 0.0038 (LLCI = 0.0001, ULCI = 0.0102). In contrast, the chain of social network interaction behaviors had a negative mediating effect value of −0.0083 (LLCI = −0.0198, ULCI = −0.0002), indicating that the overall chain exerts a negative effect on the tendency to break off kinship. The results verified H10 and H11. See Table 3 and Figure 1 for details.

### Summary of test results

Summarizing the results of the above studies, we can clearly see that the cultural variables, functional and behavioral variables specifically play a role in young people’s tendency to break off kinship. See Table 4.

**Table 4:** A summary of test results.

	Aim	Hypothesis		Results
1	Examining the relationship between cultural, functional, and behavioral variables and the tendency to break off relatives	H1	VFS → TBOF	√
		H2	Kinship support → TBOF	√
		H3	Kinship burnout → TBOF	√
		H4	SCIR → TBOF	√
		H5	AFG → TBOF	√
2	Examining the mediating effect of functional, behavioral variables between cultural Variables and the tendency to break off relatives	H6	VFS → kinship support → TBOF	√
		H7	VFS → kinship burnout → TBOF	×
		H8	VFS → SCIR → TBOF	√
		H9	VFS → AFG → TBOF	√
3	Examining the chain mediating effect of functional, behavioral variables between cultural variables and the tendency to break off relatives	H10	VFS → kinship support → kinship burnout → TBOF	√
		H11	VFS → SCIR → AFG → TBOF	√

TBOF, tendency to break off kinship; VFS, view of family-state; SCIR, scale of closely interacted relatives; AFG, activity in family groups.

## Discussion and conclusion

Based on data related to kinship interactions collected from 555 young people aged 18–35 in China, this study found through regression and chain mediation analyses that cultural perception remained the main predictor variable of whether young people adopted kinship breaking behaviors and the propensity to engage in kinship breaking, as evidenced by its comparison with the effect sizes of other variables. However, superimposing the material and psychological support and burnout from kinship relationships shows that material, emotional, and relational support from relatives can significantly reduce people’s propensity to break away, while psychological boundary violations and related life burnout increase people’s propensity to break away, and overall, this rationalism-based instrumental acquisition and burnout mediates the effect of cultural perception on whether people adopt breakaway behaviors. At the same time, the size and activity of people’s daily relative interactions based on social media significantly predicted young people’s propensity to cut off relatives. Especially, activity frequency in family groups significantly mediated the influence of cultural perception. In other words, although young people with strong family-state cultural perception are less likely to disconnect, this cultural influence also requires daily social media connections and activities to sustain and support. Of particular note in this is that it is the frequency of kinship interactions, rather than size, that plays this role. The findings of the study deserve further discussion in three main dimensions.

### Culturalism, functionalism, and behaviorism variables play a concurrent role in explaining the kinship contact status of Chinese youth, with cultural perception remaining a relatively dominant influence, while functionalism and behaviorism variables are becoming more influential

In traditional Chinese society, the attributes of agrarian society dictate that cultural perceptions based on “the differential mode of association” are the main factors influencing people’s kinship connections, as the tightness of kinship ties also directly affects how people unfold and whether they can achieve in other dimensions of social life, which is one of the reasons for the greater explanatory power of culture in the rapid development across Asia after the 1980s (Redding, 2000). However, with the expansion of China’s social transformation, i.e., from an agricultural society to an industrial society to today’s information society, the separation or even segregation of people’s workspace and birth space, and the measurement of the pros and cons of kinship from a functionalist and instrumentalist perspective has increasingly become an active or passive choice in people’s real social lives. Although the “instrumentalist differential order pattern” based on Li Peiliang still plays a role, if this role is less than the “harm” or “loss” people feel in kinship connection, the tendency of kinship disconnection will occur. Meanwhile, the development of social media has facilitated people’s kinship connections across time and space nowadays; thus, the scale and activity of people’s kinship connections in social media become factors that can significantly predict people’s tendency to connect, and these behavioral performances also significantly moderate the influence of cultural perceptions. In summary, the influence of cultural perceptions needs to exist and be maintained in behavioral interactions. The synthesis of the three theoretical perspective variables described above goes beyond Xiang’s perspective (2021) of explaining the tendency to break off relatives and weakening the impact of this phenomenon purely in terms of modern social transformation, while also integrating Hu’s meso-interpretation (2022) of the phenomenon of breaking off relatives as viewed from the perspective of intra-familial social comparisons and pressures, and at the same time Sun’s proposal (2023) advocating an explanation of this phenomenon in terms of moral culture, class mobility, and social relations. It is a continuation and synthesis of existing Chinese intellectual discussions on this issue, and it also hopes to provide a comparative case for the explanation of such phenomena in different socio-cultural contexts around the world.

### The key chain that plays a divisive role in kinship connections among Chinese youth is the push–pull effect of kinship support and burnout

Although cultural perception is the dominant variable supporting whether Chinese youth generate a propensity to disconnect or engage in disconnection behavior, our findings above also indicate whether kinship burnout outweighs the mediating effect of kinship support as a key differentiating variable in influencing young people’s decision to disconnect or not. This suggests at least two dimensions: (1) Among the factors affecting the kinship connection status of Chinese youth today, what may be most important is not how much support kinship can provide but whether the burnout caused by kinship exceeds the zero thresholds they can afford. When violations of personal boundaries, daily social comparisons, injuries, and damages reach a certain zero threshold, the impact of kinship support on the maintenance of kinship ties is overshadowed, and a kind of “nonviolent rejection” of kinship ties, which is more prevalent among youth, occurs. In other words, kinship burnout is more critical and sensitive than kinship support when speculating or predicting whether a youth will engage in kinship disconnection behavior. (2) This situation further suggests that although the inertia of cultural perceptions still supports the maintenance of kinship networks among young people in China while playing an essential supportive role in the kinship ties of young people today due to its integration of functionalist emotional, material, and relational support. However, individual boundaries, dignity, security, and fulfillment are what young people care about at the individual level. Once this boundary is violated and cumulative damage is formed, young people may disregard the role of kinship support and adopt a strategy of alienation and dilution of kinship. The situation will be further complicated by the return of local and kinship ties, as observed at the group level, where more and more young Chinese have chosen to work within the government and affiliated institutions in recent years due to COVID-19 and the international situation.

### Social media is an important venue for expressing the interaction status of young people’s relatives, and “online” relationships have become an important indicator to predict changes in young people’s kinship relationships

Since Granovetter, many indicators have been developed to understand strong and weak ties, such as temporality, emotional intensity and often classify Internet-based ties as weak and blood-based kinship ties as vital (Krackhardt, 1992). Since 2005, the rapid development of social media has led to an understanding of “relationships” from the perspective of media “infrastructure,” i.e., instead of anticipating connections through relationships, such as the closer the relationship, the more diverse the communications may be but rather anticipating relationships through “communications” (Haythornthwaite, 2002). Our study demonstrates precisely this point: whether young people maintain close contact relationships with relatives can be directly predicted by their online connection relationships and status. The power of connection size was not as good as the predictive effect of group activity, i.e., although both the number of young people’s social media connections and group activity significantly predicted their kin connection tightness and tendency to disconnect, in the mediating effects analysis, we found that connection size was not as strong as the effect of group activity on the relationship between cultural values and the disconnection effect. This means that what matters is not how many people are connected but whether they interact substantively with people in their kinship group. Relationships are only meaningful and have greater continuity when “active.”

Overall, our study provides an exploratory interpretation of kinship connections among young people in China in the current social and cultural context, i.e., cultural values are still important in sustaining kinship connections, but such connections are being affected by the increasing perception of boundaries and other impairments among young people while exploring kinship networks of young people in contemporary China needs to be examined from the perspective of social media-mediated connections. At the same time, it indicates that the current state of interpersonal relationships needs to be considered from a three-dimensional structure formed by cultural perception + functional satisfaction and burnout + social media. Especially when the state of interpersonal connections in society is closely related to the overall socio-economic development pattern of the country, it is necessary to conduct a multidimensional investigation of the interpersonal connections of Chinese youth today. It is important to note that the factors in this study are categorized into culturalism, functionalism, and behaviorism variables for explanatory clarity, mainly based on the understanding of the model, and the factors selected for each perspective can be considered more multi-dimensionally. Meanwhile, for the functionalism factors considered, kinship support and kinship burnout included three and four secondary variables, respectively. However, the choice to use moderated mediation analysis focusing on the core explanatory variables instead of structural equations in the final model analysis is worth discussing. For example, kinship support includes three dimensions of material, emotional, and relational support, but our study does not show this in detail, which needs to be presented in detail in the follow-up report. Similarly, how the moderating role of the important demographic variables of the study population plays a role in the overall model is a part that deserves additional reporting. In addition, the validity of the critical conceptual measures, such as cultural perception, which are mainly measured by the view of family-state, and the tendency to break off kinship, which is mainly represented by the gathering in the “Spring Festival” ceremony, needs to be verified in the comparison and dialogue with other studies.

## Data availability statement

The raw data supporting the conclusions of this article will be made available by the authors, without undue reservation.

## Ethics statement

Ethical review and approval was not required for the study on human participants in accordance with the local legislation and institutional requirements. Written informed consent from the patients/ participants or patients/participants legal guardian/next of kin was not required to participate in this study in accordance with the national legislation and the institutional requirements.

## Author contributions

The author confirms being the sole contributor of this work and has approved it for publication.

## Footnotes

^1^ https://www.wjx.cn/
